# Predicting Participant Compliance With Fitness Tracker Wearing and Ecological Momentary Assessment Protocols in Information Workers: Observational Study

**DOI:** 10.2196/22218

**Published:** 2021-11-12

**Authors:** Gonzalo J Martinez, Stephen M Mattingly, Pablo Robles-Granda, Koustuv Saha, Anusha Sirigiri, Jessica Young, Nitesh Chawla, Munmun De Choudhury, Sidney D'Mello, Gloria Mark, Aaron Striegel

**Affiliations:** 1 Computer Science and Engineering University of Notre Dame Notre Dame, IN United States; 2 Thomas M Siebel Center for Computer Science University of Illinois Urbana-Champaign Urbana, IL United States; 3 Microsoft Research Montreal, QC Canada; 4 School of Interactive Computing Georgia Institute of Technology Atlanta, GA United States; 5 Indian School of Business Gachibowli Hyderabad Telangana India; 6 Center for Research Computing University of Notre Dame Notre Dame, IN United States; 7 Institute of Cognitive Science University of Colorado Boulder Boulder, CO United States; 8 Informatics Department University of California, Irvine Irvine, CA United States

**Keywords:** adherence, compliance, wearables, smartphones, research design, ecological momentary assessment, mobile sensing, mobile phone

## Abstract

**Background:**

Studies that use ecological momentary assessments (EMAs) or wearable sensors to track numerous attributes, such as physical activity, sleep, and heart rate, can benefit from reductions in missing data. Maximizing compliance is one method of reducing missing data to increase the return on the heavy investment of time and money into large-scale studies.

**Objective:**

This paper aims to identify the extent to which compliance can be prospectively predicted from individual attributes and initial compliance.

**Methods:**

We instrumented 757 information workers with fitness trackers for 1 year and conducted EMAs in the first 56 days of study participation as part of an observational study. Their compliance with the EMA and fitness tracker wearing protocols was analyzed. Overall, 31 individual characteristics (eg, demographics and personalities) and behavioral variables (eg, early compliance and study portal use) were considered, and 14 variables were selected to create beta regression models for predicting compliance with EMAs 56 days out and wearable compliance 1 year out. We surveyed study participation and correlated the results with compliance.

**Results:**

Our modeling indicates that 16% and 25% of the variance in EMA compliance and wearable compliance, respectively, could be explained through a survey of demographics and personality in a held-out sample. The likelihood of higher EMA and wearable compliance was associated with being older (EMA: odds ratio [OR] 1.02, 95% CI 1.00-1.03; wearable: OR 1.02, 95% CI 1.01-1.04), speaking English as a first language (EMA: OR 1.38, 95% CI 1.05-1.80; wearable: OR 1.39, 95% CI 1.05-1.85), having had a wearable before joining the study (EMA: OR 1.25, 95% CI 1.04-1.51; wearable: OR 1.50, 95% CI 1.23-1.83), and exhibiting conscientiousness (EMA: OR 1.25, 95% CI 1.04-1.51; wearable: OR 1.34, 95% CI 1.14-1.58). Compliance was negatively associated with exhibiting extraversion (EMA: OR 0.74, 95% CI 0.64-0.85; wearable: OR 0.67, 95% CI 0.57-0.78) and having a supervisory role (EMA: OR 0.65, 95% CI 0.54-0.79; wearable: OR 0.66, 95% CI 0.54-0.81). Furthermore, higher wearable compliance was negatively associated with agreeableness (OR 0.68, 95% CI 0.56-0.83) and neuroticism (OR 0.85, 95% CI 0.73-0.98). Compliance in the second week of the study could help explain more variance; 62% and 66% of the variance in EMA compliance and wearable compliance, respectively, was explained. Finally, compliance correlated with participants’ self-reflection on the ease of participation, usefulness of our compliance portal, timely resolution of issues, and compensation adequacy, suggesting that these are avenues for improving compliance.

**Conclusions:**

We recommend conducting an initial 2-week pilot to measure trait-like compliance and identify participants at risk of long-term noncompliance, performing oversampling based on participants’ individual characteristics to avoid introducing bias in the sample when excluding data based on noncompliance, using an issue tracking portal, and providing special care in troubleshooting to help participants maintain compliance.

## Introduction

### Background

In the past decade, an increasing variety of sensors have been used as research tools, such as smartphones [1-9], wearables [9-19], ecological momentary assessments (EMAs) [20-23], social media [24,25], and other sensing modalities [26-30]. However, the effectiveness of these studies depends on the completeness of the data generated, which further relies on participant compliance. When designing an observational study, the most important factor for ensuring quality data is compliance (sometimes referred to as adherence [31]).

Typically, compliance decreases throughout the life of a study [1]. Compliance has been found to be as low as 16% by the end of an almost year-long study [32], and it varies considerably from 80% to between 10% and 20% during certain periods in the study [33]. This pattern persists in shorter studies as well. For example, in the study of wearable compliance by Evenson et al [34], it was found that only 78% of >15,000 participants completed at least 21% of the possible data collection. This can considerably reduce the sample size available for analysis, should continuous measurements be necessary. For instance, despite enrolling 646 participants, Wang et al [35] only analyzed data from 159 participants because of the lack of compliance. Finally, other studies have found compliance to be related to participant characteristics [12,13,31,36], which increases the odds of introducing bias when excluding participants from analysis because of noncompliance [37]. Therefore, we posit that the ability to identify the compliance of individuals early and resolve issues that can affect study participation would be invaluable to the research community. Prior works have found associations between certain participant characteristics that could be used to predict compliance early in a study, although the evidence is conflicting.

Early works in the field by Schüz et al [22] and Courvoisier et al [38] suggested that compliance with EMAs was not associated with participant characteristics. Specifically, Schüz et al [22] administered random prompts for EMAs in a 6-day study of 119 smokers and found no association between EMA compliance and smoking habits, race, sex, education level, or marital status. Similarly, Courvoisier et al [38] found no associations between a phone call–based EMA monitoring protocol and sex, age, education level, linguistic region, life satisfaction, or personality. However, more recent studies have found conflicting evidence. Dzubur et al [39] examined EMA responses and reported approximately 80% compliance, but compliance was influenced by the participant-level factors of income and ethnicity; lower-income or Hispanic mothers were less likely to respond to surveys. Finally, in a meta-analysis on factors that contribute to EMA compliance administered from smartphones or with wearables for those aged <18 years, Wen et al [36] reported a general response rate of approximately 78% to surveys, which varied based on clinical status; those without disorders had lower response rates with more prompts (≥6 times: 75%; 2-3 times: 92%), whereas those with a clinical status responded more often to increased prompts (≥6 times: 89%; 2-3 times: 74%). These papers suggest that participant-level factors such as income, ethnicity, and clinical status can interact with compliance in longitudinal studies using EMAs.

Several studies using wearable accelerometers have found associations between compliance and various participant characteristics, such as income, age, smoking, and having tertiary education [12,13,34]. A 4-day study involving 3601 participants [13] found that higher compliance, defined as wearing time, was associated with being older, not smoking, and having a full-time job, tertiary education, and high self-reported health, whereas no associations were found with income level or sex. As noted earlier, the study by Evenson et al [34] defined compliance as wearing an accelerometer for 10 hours a day, 3 days out of 6 days, in a study of 15,153 participants in a Hispanic community. The study reported higher compliance for those participants who are married or partnered; those with higher household income; those who are male, older, and employed or retired; those not born in the United States; those preferring Spanish over English; and those having a lower BMI. Similarly, a repeated measures study of adolescent females that deployed accelerometers 7 months apart found that physical activity level and race were associated with compliance [12]. The same study found that compliance was trait-like; higher compliance in the first session was associated with higher compliance in subsequent redeployments of accelerometers 7 months apart.

Recent studies that included smartwatches, fitness trackers, or smartphones have also found participant characteristics to be related to compliance [31-33,40]. Harari et al [33] analyzed 3 student population samples to understand participants’ motivations for self-tracking using passive sensing and active logging in relation to compliance. The study correlated participant characteristics with behaviors that motivated self-tracking and found that more agreeable, younger, and extroverted participants had increased productivity and health behaviors that were positively correlated with compliance. In addition, the study found that neuroticism, openness, and being female were correlated with well-being and daily activities motivation to self-track, which were in turn positively correlated with compliance. No significant correlations were found with conscientiousness and well-being measures. On the other hand, in a 4-year study of 698 college students where compliance was studied as a binary variable, it was found that extraversion and openness negatively correlated with compliance, whereas conscientiousness and agreeableness positively correlated with compliance, and neuroticism did not significantly correlate with compliance [31]. In addition, the study reported that the first month of compliance correlated with whole study compliance, a result in line with Rowlands et al [12].

Jeong et al [18] studied how individuals perceive their study participation in a sample of 50 students using Apple Watch and found that limitations of the devices themselves or personal reasons could get in the way of study participation and cause noncompliance. The study found that when participants needed to charge the devices during the night or at least once a day, compliance decreased. Similarly, participants would forget to wear the smartwatch depending on certain situations, such as staying at home for the weekend or going out with friends. However, the reported patterns of wearing behavior do not necessarily match those of modern wearables where battery life lasts multiple days or those of a working population [41].

### Objective

In summary, there is extensive literature showing that participant characteristics and compliance are related, albeit with conflicting results. However, there remain several issues before these findings could be used in practice. For instance, several works define compliance as a binary variable with two outcomes depending on a specific definition that is not universally applicable, for example, in ≥80% [31], 10 hours a day for 3 out of 7 days [34], or >16 hours a day for 7 days [12]. The use of such specific and inconsistent definitions of compliance makes it challenging to apply the findings from other studies with different thresholds to meet the requirements of a new study. In addition, most existing works do not provide any metric of model fit or error that provides guidance on the predictive power of the models, with the exceptions of Lee et al [13] reporting *R*^2^=0.03 and Hermsen et al [32] reporting *R*^2^=0.099. Existing works rely only on a training set of data, that is, no testing set was used to report predictive performance. This means that we cannot know beforehand if participant characteristics could be effective in predicting compliance before a study starts or early on. Given these limitations, the objective of our paper is to address the following research questions (RQs) using a generalizable definition of compliance and considering personal characteristics that are commonly or easily assessed in other studies:

- RQ1: To what extent can personal characteristics measured before the start of a study predict long-term compliance?
- RQ2: How does early assessment of compliance (ie, during the first 2 weeks of study participation) predict future compliance?
- RQ3: Are participants’ perceptions of study participation, feedback, and issue reporting correlated with compliance?

## Methods

We used our Tesserae [42] study—a 1-year, large-scale, multimodal study of working professionals with a rich set of psychological and health-related data to extract meaningful variables that explain variations in compliance.

### Participants and Recruitment

**Figure 1:**
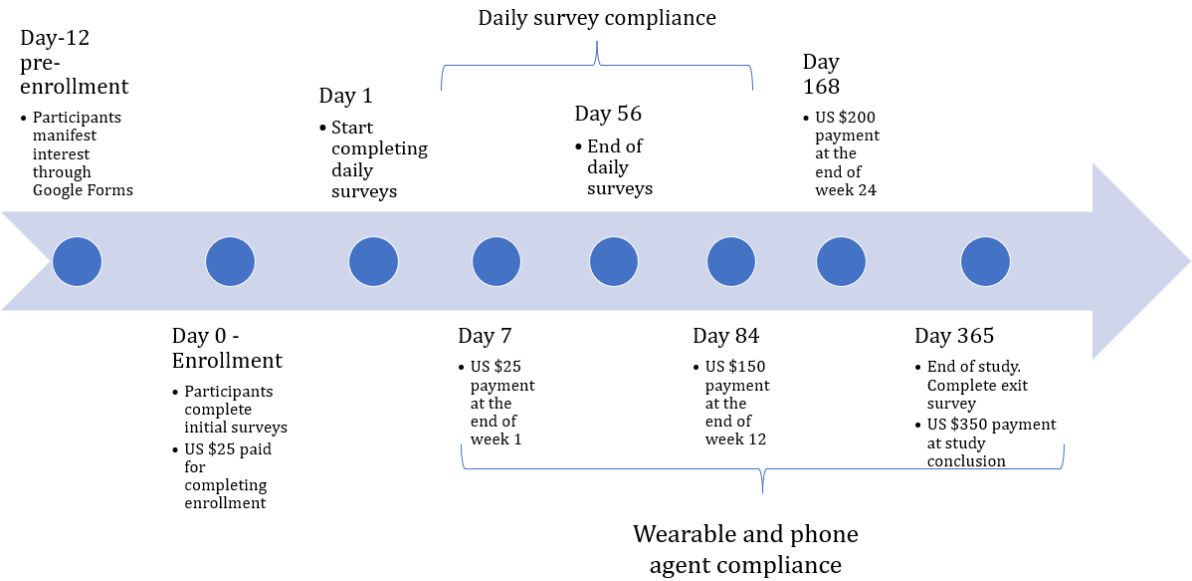
Timeline of study participation.

Tesserae recruited 757 participants from cognitively demanding professions (eg, information workers) to participate in a 1-year study exploring the extent to which widely available sensing streams could predict various individual difference variables and job performance dimensions. As such, the study was observational in nature and did not implement interventions beyond interacting with participants to resolve participation issues. Individuals were drawn from throughout the United States. Participants were enrolled both in person and remotely from January 2018 to July 2018. The study concluded data gathering in mid-April 2019. A timeline of the study can be found in Figure 1. Participants were divided into 2 sets: blinded and nonblinded (Table 1). Responses to initial and daily surveys from the blinded set were withheld from researchers by the study sponsor until the end of phase 1 of the multimodal objective sensing to assess individuals with context [43] program in May 2020. Researchers had full access to participant data in the nonblinded set.

**Table 1:** Cohort distribution in the blinded and nonblinded samples after data preprocessing. A chi-square test of independence showed that the cohort distribution is different between the blinded and nonblinded samples (χ^2^(4)=129.53; *P*<.001).

Cohort	Blinded, n (%)	Nonblinded, n (%)
Large multinational technology services firm	42 (28)	249 (41.7)
Large midwestern technology or engineering firm	34 (22.7)	144 (24.1)
Midwestern software engineering firm	5 (3.3)	21 (3.5)
Midwestern university	57 (38)	32 (5.4)
Various other companies	12 (8)	151 (25.3)
Total	150 (100)	597 (100)

### Procedures

Individuals participating in the study were provided with a wearable (Garmin vivoSmart 3); a smartphone agent (phone app) derived from StudentLife [2]; and a set of Bluetooth beacons to demarcate home, work, and proximity to others in the study and were requested to provide read access to social media (Facebook and Twitter) [44]. An initial set of psychological and health-related surveys were collected (Multimedia Appendix 1 [45-57]) at enrollment in the study. In addition, short, daily versions of many of the aforementioned surveys were administered, as well as context, current stress level, and current anxiety level assessments. Daily surveys were administered via the Qualtrics Experience Management platform, prompted by an SMS text message, designed to be answered in <2 minutes, and with a 4-hour window for completion. Users received daily survey prompts at either 8 AM, noon, or 4 PM during the initial 8 weeks (56 days) of a participant’s year in the study. Participant compliance and troubleshooting were provided through a user-facing web-based portal and managed by the study personnel. In the case of data from participants belonging to the blinded set, researchers did not send the surveys or receive the responses directly. These surveys were administered by the study sponsor and stored apart from nonblinded data, allowing researchers the use of nonblinded data for exploratory analyses while still having a separate sample to test out-of-sample performance.

The compliance rate for the study was tracked for 3 of the major sensing streams: daily surveys, wearables, and phone agents, which was not analyzed (see the *Measures* section). Bluetooth beacon compliance was not tracked as an individual could be complying without being in the range of a beacon for the study. Conversely, social media compliance was not tracked, as it was an opt-in sensor and only required one-time authorization.

Participants were compensated based on their compliance. Participants could continuously review their compliance and report issues through a dynamic web-based portal. On the basis of cohort requirements, participants were either paid a stipend or entered into a weekly lottery. For stipend participants, those with average compliance of at least 80% across all streams could receive up to US $750 at the completion of the study, broken up as shown in Figure 1. Lottery participants received a ticket per day for each compliant (>80%) stream (wearable, phone agent, and daily survey). A US $250 weekly lottery was held for every 25 participants.

Experimenters could increase compliance in one or more streams at their discretion because of changes in circumstances that precluded compliance for a limited time. For example, participants whose wearables broke and who reported it to researchers received full compliance for the wearable stream until they received a replacement wearable. In the duration of the study, 325 wearable device replacements were issued [41]. Other examples include international travel, which prohibited SMS text message receipt of the daily survey; a damaged or replaced cell phone; or change of carrier that affected phone agent compliance and the receipt of daily surveys. If the participants did not inform the researchers regarding such problems related to their sensing streams, they received zero compliance in that period.

Periodic reminders were sent every week via email to participants exhibiting noncompliance (missing recent data or cumulative noncompliance). Participants could decide to stop participating at any point in the study. In addition, participants exhibiting continued noncompliance without response over multiple months (≥3) were considered ineligible for subsequent rewards and were excluded from the study. In total, 107 participants were considered to have dropped out. Nevertheless, although these participants were considered to have dropped from the Tesserae study, they were still considered in the analyses in this work.

### Measures

#### Compliance

Daily survey compliance was defined as the response rate, that is, the number of surveys responded to over the number of surveys sent. Not receiving a prompt because of the phone being turned off or being out of coverage was not differentiated from receiving and not responding to a prompt; both were considered noncompliant. Wearable compliance was computed in 30-minute nonoverlapping windows, whereby an individual was considered to have been compliant if any wearable data from any stream of the fitness tracker (eg, heart rate, step count, or physical activity) was recorded within that window. However, considering the heart rate or the combination of all sensor streams with the Garmin vivoSmart 3 leads to differences in the calculation of compliance of <1% [41]. Therefore, wearable compliance is the number of 30-minute windows with any data over the number of 30-minute windows in the study, with 48 windows a day for up to 365 days.

**Figure 2:**
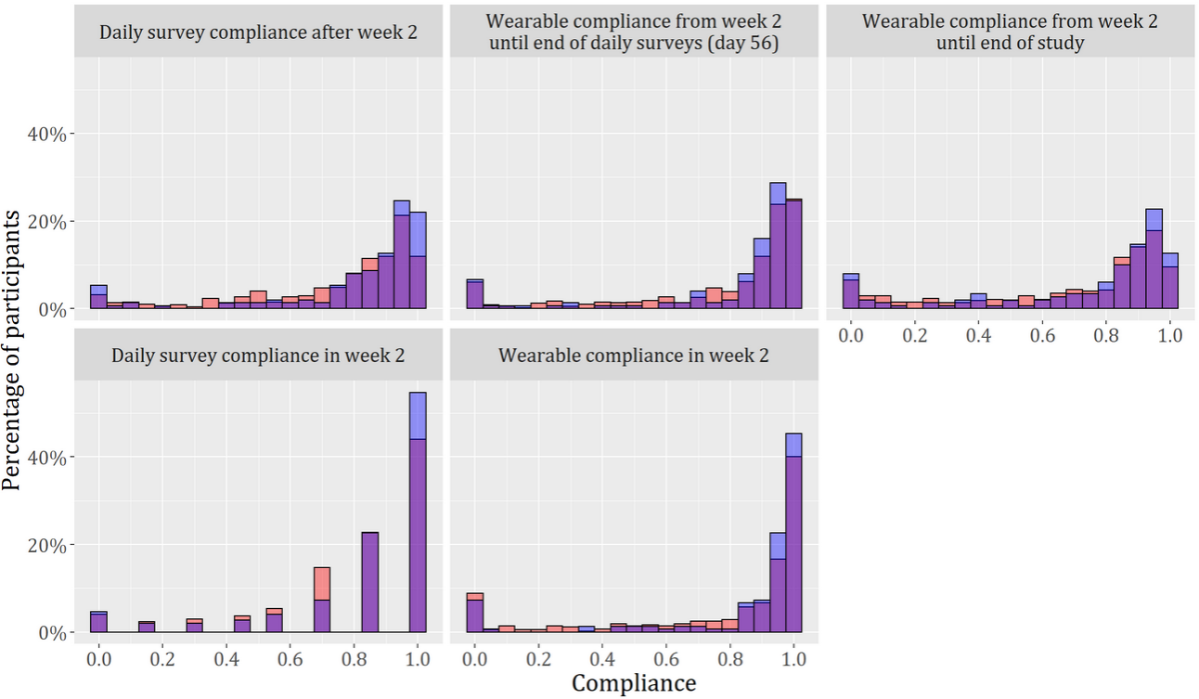
Distribution of compliance in the nonblinded set in red (n=597) and the blinded set in blue (n=150). The superposition of both sets is in purple.

Participants were requested to wear the device 24-7 to capture their sleep and daily activities. The roughly 5-day battery life and rapid charge rate allowed a brief charging window each day to not exhaust the wearable battery [41]. Given that the first 2 weeks of compliance would be used to predict long-term compliance (RQ2), we discarded these 2 weeks from the dependent variables and calculated them starting at week 3. We referred to these simply as *wearable compliance* and *survey compliance*, whereas the variables used as predictors were referred to as *wearable compliance in week 2* and *survey compliance in week 2* (see Figure 2 and Table 2 for compliance distributions and correlations).

**Table 2:** Pearson correlation (*r*) of compliance in the nonblinded (below the diagonal) and blinded sets (above the diagonal).

	Daily survey compliance after week 2	Wearable compliance after week 2	Daily survey compliance in week 2	Daily wearable compliance in week 2
Daily survey compliance after week 2	1	0.79	0.81	0.69
Wearable compliance after week 2	0.73	1	0.60	0.72
Daily survey compliance in week 2	0.69	0.51	1	0.58
Daily wearable compliance in week 2	0.56	0.61	0.54	1

Similar to wearable compliance, phone agent compliance was calculated in half-hour time windows. However, the phone agent was intended to run without any user input or action apart from the initial installation and updates. This presented several challenges for researchers, as participants used 112 different models of mobile devices from 14 manufacturers throughout the study. The main challenges were issues of high battery use, failure to run continuously in the background, and an initial lack of feedback to participants regarding whether data were being collected. As a result, missing data more likely reflected the researchers’ technical ability to keep the app running on all devices rather than user characteristics or behaviors. Therefore, we decided *not* to consider phone agent compliance rates.

#### Demographics, Psychological Traits, and Health-Related Characteristics

The shared Tesserae demographics, psychological, and behavioral data set comprised 31 variables that were collected during the study and could be used as predictors. These variables can be categorized as demographics, personality [45], anxiety [46], affect [47], health [48-52], cognitive ability [53], job performance [54-57], and behavior characteristics collected through the study website [42], such as log-ins and issue tickets submitted. Psychometrically validated inventories were used to collect all survey-based measures, except for demographics (Table 3).

**Table 3:** Demographic questions asked at the onset of study participation.

Item	Question	Options
Age	How old are you?	Any number
Sex	Are you male or female?	Male Female
Household income level	In which of these groups did your total household income (from all sources) fall in 2016?	<US $25,000 US $25,000 to US $49,999 US $50,000 to US $74,999 US $75,000 to US $99,999 US $100,000 to US $124,999 US $125,000 to US $150,000 >US $150,000
Supervise	Think about your main job. Do you supervise or manage anyone in this job?	Yes No
English as native language	Is English your native language?	Yes No
Education level	What is your highest level of education?	Some high school (or equivalent) High school degree (or equivalent) Some college College degree Some graduate school Master’s degree Doctoral degree, such as a PhD, MD, or JD
Had a wearable	Do you currently use a wearable like a Fitbit or other fitness device?	Yes No

### Data Exclusion and Preprocessing

**Figure 3:**
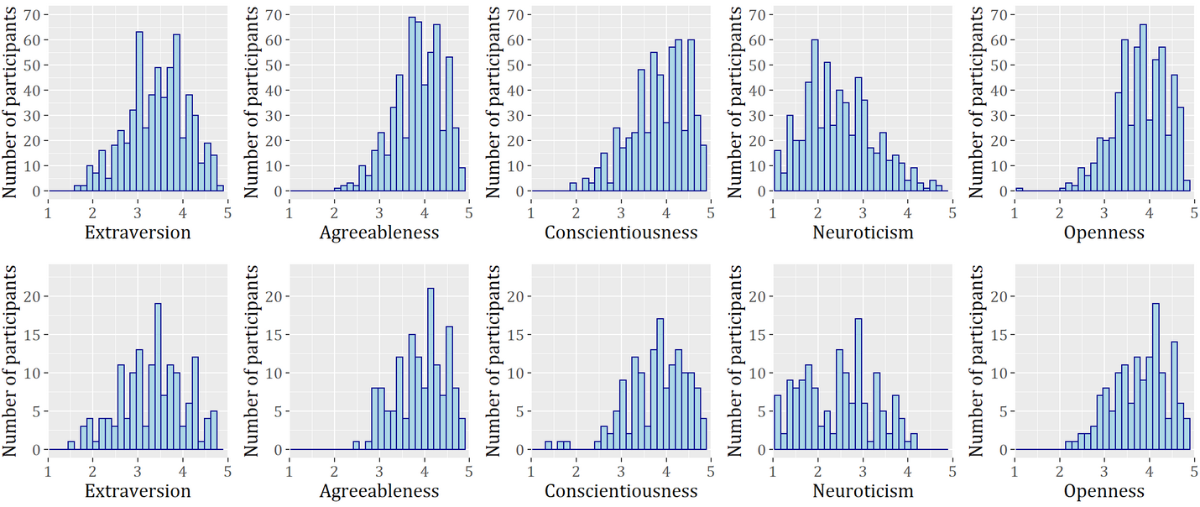
Distribution of personalities in the nonblinded set (top) and blinded set (bottom). Independent sample *t* tests found no significant differences between the two sets across personalities (all *P* values >.09).

Most of the predictors were obtained from the initial survey conducted during enrollment. Variables in the blinded and nonblinded samples were treated in the same manner. The variables for household income and education were relabeled to address class imbalance. Classes of <US $25,000 and from US $25,000 to US 49,999 were merged into one class (<US $50,000), whereas the classes US $100,000 to US $124,999 and US $125,000 to US $150,000 were merged into a single class as well (US $100,000 to US $150,000). In the case of education, the classes were coalesced into *no college degree*, *college degree,* and *graduate degree.* A total of 32 respondents did not answer the wearable ownership question. To avoid discarding these observations, we generated an additional *unknown* category. All other categorical variables <5 missing observations. Missing survey data were excluded. After data preprocessing, our nonblinded sample contained 597 participants, and our blinded sample contained 150 participants. The participants’ demographics are available in Table 4. The distribution of participants’ personality variables are available in Figure 3.

**Table 4:** Demographics of the blinded set and nonblinded set participants. Results of chi-square tests of independence are shown in the table.

Demographics		Nonblinded (n=597)	Blinded (n=150)	Chi-square (*df*)	*P* value
**Age (years)**					
	Values, mean (SD)	34.33 (9.37)	37.18 (10.83)	N/A^a^	N/A
	Values, range	21-68	20-63	N/A	N/A
**Sex, n (%)**					
	Male	346 (58)	93 (62)	0.7 (1)	.42
	Female	251 (42)	57 (38)	0.7 (1)	.42
**Income (US $), n (%)**					
	<49,999	46 (7.7)	7 (4.7)	4.8 (4)	.31
	50,000-74,999	126 (21.1)	34 (22.7)	4.8 (4)	.31
	75,000-99,999	129 (21.6)	33 (22.0)	4.8 (4)	.31
	100,000-150,000	162 (27.1)	50 (33.3)	4.8 (4)	.31
	150,000	134 (22.4)	26 (17.3)	4.8 (4)	.31
**Education, n (%)**					
	No college degree	43 (7.2)	9 (6)	0.3 (2)	.86
	College degree	326 (54.6)	84 (56)	0.3 (2)	.86
	Graduate degree	228 (38.2)	57 (38)	0.3 (2)	.86
**Supervisor role, n (%)**					
	Nonsupervisor	313 (52.4)	88 (58.7)	1.6 (1)	.20
	Supervisor	284 (47.6)	62 (41.3)	1.6 (1)	.20
**English as first language, n (%)**					
	No	86 (14.4)	8 (5.3)	8.2 (1)	.004
	Yes	511 (85.6)	142 (94.7)	8.2 (1)	.004
**Had a wearable, n (%)**					
	No	277 (46.4)	63 (42)	1.2 (2)	.56
	Yes	292 (48.9)	78 (52)	1.2 (2)	.56
	Unknown	28 (4.7)	9 (6)	1.2 (2)	.56

^a^N/A: not applicable.

### Analyses

#### Addressing RQ1 and RQ2

##### Exploratory Analysis and Variable Selection

We conducted exploratory analyses on the nonblinded set to determine whether variables that were not studied before in the context of compliance or without enough supporting theory behind them are related to compliance, that is, job performance, anxiety, sleep quality, affect, smoking and alcohol use, and physical activity. We compiled the results of our exploratory analysis, show the distribution of other variables in Multimedia Appendix 1, and show a model including all available variables in Multimedia Appendix 2 [12,13,22,52,58-67] along with the interpretation of these variables and their possible relationship with compliance. As these variables, as well as statistics from our study portal use, are uncommon and specific to our study, we conducted our main analyses with a reduced set of variables (14/31, 45%) comprising demographics, personality, and early compliance. We considered our reduced set to be generalizable to more studies (eg, age as opposed to log-ins to our study portal) and to be of interest based on related work. Table 5 shows the means and SDs of the selected variables.

**Table 5:** Means and SDs of continuous variables in the models. Independent sample t tests (2-tailed) show no evidence of differences in the means between the nonblinded and blinded data sets in all variables tested, except survey compliance and wearable compliance in week 2.

Variable	Nonblinded, mean (SD)	Blinded, mean (SD)	*t* test (*df*)	*P* value
Age (years)	34.33 (9.37)	37.18 (10.83)	—^a^	—
Extraversion	3.45 (0.67)	3.34 (0.73)	1.60 (218)	.11
Agreeableness	3.88 (0.56)	3.93 (0.58)	−0.99 (225)	.33
Conscientiousness	3.89 (0.66)	3.85 (0.68)	0.69 (224)	.49
Neuroticism	2.46 (0.78)	2.46 (0.83)	0.03 (220)	.98
Openness	3.82 (0.60)	3.82 (0.63)	−0.01 (222)	>.99
Number of log-ins in week 2	0.40 (0.94)	0.34 (0.67)	0.82 (312)	.41
Number of issues in week 2	0.00 (0.01)	0.00 (0.00)	1.32 (292)	.19
Survey compliance	0.75 (0.26)	0.80 (0.26)	−2.18 (229)	.03
Wearable compliance	0.68 (0.32)	0.73 (0.31)	−1.54 (234)	.12
Survey compliance in week 2	0.80 (0.27)	0.84 (0.27)	−1.50 (229)	.13
Wearable compliance in week 2	0.78 (0.32)	0.85 (0.28)	−2.45 (256)	.01

^a^The samples of age in each set were not normal (Shapiro-Wilk test: *P*<.001) and appeared as not drawn from the same distribution (Kolmogorov-Smirnov test: *P*=.01). Therefore, the differences in age were not assessed with *t* tests.

Among the demographic measures collected (Table 3), 2 measures are unique to our data set: previous wearable ownership and having a supervising role. Previous wearable ownership is of special interest because an association with compliance could point to a useful characteristic that could be applied to other populations as well. This variable can contribute in contrasting ways to wearable compliance. On the one hand, one can expect this variable to positively affect wearable compliance, as those with prior wearable experience may be familiar with wearable requirements and capabilities. On the other hand, prior work found that those who did not own a smartwatch and received one in a study could feel motivated to be more diligent and have higher wearable compliance [18]. In addition, we included *supervisory role* in examining compliance. A supervisory role may indicate a busy life or schedule that impairs the ability to participate successfully in the study. However, this variable only applies to a working population.

##### Model Creation

The response variables in our models are, by definition, ratios with possible values constrained to the interval from 0 to 1 (inclusive). Given our intention of having interpretable models and that our response variables are proportions (windows that contained data over total windows and response rate), we decided to create the models using the beta regression model proposed by Ferrari et al [68], which was specifically designed to model rates and proportions using the beta distribution. We relied on the flexibility of the beta distribution to take different shapes and to represent probabilities and proportions and assumed that the beta distribution would be able to represent distributions that are not normally distributed, such as those in Figure 2 (lack of normality further confirmed through Shapiro-Wilk [69] test *P*<.001). However, the beta regression model cannot handle values of exactly 0 or 1. Thus, we transformed the dependent variables using the following equation [70], with y being the response variable:



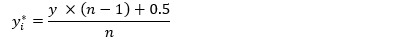



For example, the responses of 0 and 1 were transformed to 0.0008 and 0.9992, respectively.

Using 14 variables as predictors, we created hierarchical beta regression models that addressed RQ1 and RQ2. Specifically, to address RQ1, we created model 1s (s=survey as dependent variable) and model 1w (w=wearable as dependent variable), including demographics and personality measures as predictors that can be assessed before starting the study and formally enrolling participants. To address RQ2, we created 4 different models. Models 2s/2w added survey compliance in week 2, which entailed a greater effort to collect than a single survey; however, it did not require giving a wearable to participants while allowing the capture of trait-like compliance. Models 3s/3w added in daily wearable compliance in week 2, which implies that to be able to measure all the predictors, participants would have to have a wearable device for 2 weeks. Note that early compliance variables are entered in the model as percentages to obtain the OR when there is a 1% change in early compliance.

##### Model Evaluation

As the models contained a different number of variables and *R^2^* can be inflated because of overfitting of the data, we computed the Akaike information criterion and conducted likelihood ratio tests to compare models trained in the nonblinded data set. We computed *R^2^*, root mean square error, and mean absolute error (MAE) with 5-fold cross-validation to ensure that models trained in a reduced set of the same data have a good fit, have low prediction error, and are robust and unchanging, given more or less information. Finally, we assessed the out-of-sample performance of the model on the blinded data set that was not used (or seen) during modeling.

Given the multiple comparisons involved in our models, all the *P* values of the predictors in each model presented in the results were adjusted using the false discovery rate [71] correction in the stats package [72]. We interpreted the results when the adjusted *P* values in the models were <.10. Multicollinearity was assessed using the generalized variance inflation factors [73]. Visual inspection of diagnostic plots, such as residuals versus indices of observations, Cook distance plot, and residuals versus linear predictor, was conducted following the recommendations of Ferrari et al [68].

#### Addressing RQ3

A total of 623 individuals completed an *optional* assessment of participation in the study (see Table 6 for inventory and responses). The 5-point Likert scale items were scored from 1 to 5, and nonresponses were dropped. Responses were correlated with compliance in the study. Note that although the items asked in this survey relate to study participation, they were not used for modeling compliance, given that they were asked at the end of the study (RQ1 and RQ2).

**Table 6:** Exit survey questions related to compliance (N=623 participants answered the exit survey in Tesserae).

Question and item scale and options		Percentage of responses, n (%)^a^	Values, mean (SD)	Total responses, n
**How difficult was it to participate in our study?**				
	1–Extremely easy	215 (37.3)	1.90 (0.89)	576
	2–Somewhat easy	251 (43.6)	1.90 (0.89)	576
	3–Neither easy nor difficult	71 (12.32)	1.90 (0.89)	576
	4–Somewhat difficult	35 (6.1)	1.90 (0.89)	576
	5–Extremely difficult	4 (0.7)	1.90 (0.89)	576
**What were the biggest difficulties in maintaining compliance? (Select all that apply)^b^**				
	Technical issues (eg, device broke and did not work)	280 (50.5)	N/A^c^	555
	Personal reasons (eg, friends, family, sickness, injury, and travel)	203 (36.6)	N/A	555
	Work reasons (eg, change of employment, promotion, and busy schedule)	107 (19.2)	N/A	555
	Survey issues (eg, surveys too often, too long, or too many)	75 (13.5)	N/A	555
	Privacy issues (eg, type or quantity of data collected and worries about data safety)	20 (3.6)	N/A	555
	Difficulty with setup	48 (8.6)	N/A	555
	Other—please describe	63 (11.4)	N/A	555
**Was your compensation adequate for your participation in the study?**				
	1–Extremely inadequate	11 (1.9)	4.06 (1.02)	577
	2–Somewhat inadequate	46 (8.0)	4.06 (1.02)	577
	3–Neither adequate nor inadequate	81 (14.0)	4.06 (1.02)	577
	4–Somewhat adequate	194 (33.6)	4.06 (1.02)	577
	5–Extremely adequate	244 (42.3)	4.06 (1.02)	577
**How useful was the compliance portal?**				
	I do not remember using the portal	51 (8.9)	N/A	576
	Strongly disagree	17 (3.0)	N/A	576
	Somewhat disagree	33 (5.7)	N/A	576
	Neither agree nor disagree	100 (17.4)	N/A	576
	Somewhat agree	227 (39.4)	N/A	576
	Strongly agree	148 (25.7)	N/A	576
**If you had issues, how satisfied were you with the timely resolution of any issues?**				
	1–Extremely dissatisfied	4 (0.7)	4.37 (0.88)	573
	2–Somewhat dissatisfied	22 (3.8)	4.37 (0.88)	573
	3–Neither satisfied nor dissatisfied	62 (10.8)	4.37 (0.88)	573
	4–Somewhat satisfied	155 (27.1)	4.37 (0.88)	573
	5–Extremely satisfied	329 (57.4)	4.37 (0.88)	573
**Can we contact you if we run another study like this?**				
	Yes	548 (95.1)	N/A	576
	No	28 (4.9)	N/A	576

^a^All percentages are calculated over the number of people who completed the item.

^b^Percentages do not sum up to 100% because more than 1 option is allowed.

^c^N/A: not applicable.

## Results

### Overview

The details and performance of the models addressing RQ1 and RQ2 are presented in Tables 7-9. To ensure that the models were valid, we followed the diagnostic tests outlined in the previous section. We calculated (generalized variance inflation factors) values for all models and found that all values were ≤1.27. Therefore, it is reasonable to assume that multicollinearity is not an issue. Diagnostic plots also did not raise any issues.

**Table 7:** Model descriptions for beta regression models trained on the nonblinded set predicting survey compliance.^a^

Category variables				Survey compliance										
				Model 1s				Model 2s				Model 3s		
				OR^b^ (95% CI)		*P* value		OR (95% CI)		*P* value		OR (95% CI)		*P* value
Intercept^c^				1.40 (0.05 to 2.76)		.11		−1.50 (−2.73 to −0.26)		.07		−1.74 (−2.95 to −0.54)		*.02*
**Demographics**														
	Age (years)		1.02 (1.00 to 1.03)		*.03*		1.01 (1.00 to 1.02)		.17		1.01 (1.00 to 1.02)		.39	
	Sex (male)		1.04 (0.70 to 1.27)		.91		1.12 (0.94 to 1.33)		.40		1.09 (0.92 to 1.30)		.49	
	**Income (US $)**													
		50,000-75,000	1.04 (0.70 to 1.53)		.97		0.95 (0.68 to 1.34)		.82		0.81 (0.58 to 1.14)		.42	
		75,000-100,000	0.97 (0.65 to 1.43)		.97		0.89 (0.63 to 1.26)		.69		0.79 (0.56 to 1.11)		.39	
		100,000-150,000	0.9 (0.61 to 1.33)		.81		0.93 (0.66 to 1.32)		.78		0.82 (0.59 to 1.16)		.44	
		≥150,000	0.67 (0.44 to 1.01)		.13		0.73 (0.50 to 1.05)		.23		0.65 (0.45 to 0.93)		.*06*	
	Supervisor (yes)		0.65 (0.54 to 0.79)		*<.001*		0.81 (0.68 to 0.96)		*.07*		0.81 (0.69 to 0.96)		*.05*	
	English (as first language)		1.38 (1.05 to 1.80)		*.06*		1.15 (0.91 to 1.45)		.43		1.06 (0.84 to 1.33)		.74	
	**Education level**													
		College degree	0.83 (0.57 to 1.20)		.49		0.91 (0.66 to 1.27)		.69		0.92 (0.67 to 1.26)		.74	
		Graduate degree	1.01 (0.69 to 1.48)		.97		1.13 (0.80 to 1.58)		.69		1.11 (0.80 to 1.55)		.76	
	**Had a wearable**													
		Unknown	1.45 (0.94 to 2.25)		.17		1.12 (0.76 to 1.64)		.69		1.04 (0.71 to 1.52)		.88	
		Yes	1.25 (1.04 to 1.51)		*.06*		1.13 (0.96 to 1.34)		.29		1.05 (0.89 to 1.24)		.73	
**Personality**														
	Extraversion		0.74 (0.64 to 0.85)		*<.001*		0.82 (0.72 to 0.93)		*.02*		0.84 (0.74 to 0.96)		*.04*	
	Agreeableness		0.85 (0.70 to 1.02)		.16		0.93 (0.79 to 1.10)		.64		0.99 (0.84 to 1.16)		.89	
	Conscientiousness		1.25 (1.07 to 1.46)		*.02*		1.25 (1.09 to 1.42)		*.01*		1.26 (1.11 to 1.44)		*.003*	
	Neuroticism		0.9 (0.79 to 1.03)		.20		0.90 (0.80 to 1.02)		.23		0.91 (0.81 to 1.02)		.31	
	Openness		0.99 (0.85 to 1.16)		.97		1.00 (0.87 to 1.14)		.95		0.97 (0.85 to 1.11)		.75	
**Behavior**														
	Survey compliance in week 2 (%)		—^d^		—		1.03 (1.03 to 1.04)		*<.001*		1.02 (1.02 to 1.03)		*<.001*	
	Wearable compliance in week 2 (%)		—		—		—		—		1.01 (1.01 to 1.01)		*<.001*	

^a^*P* values of <.10 are denoted in italics.

^b^OR: odds ratio.

^c^Intercept is not an odds ratio but an estimate.

^d^Variable was not included in the model.

**Table 8:** Model descriptions for beta regression models trained on the nonblinded set predicting wearable compliance (from least to most effort in data collection).^a^

Category variables				Wearable compliance											
				Model 1w				Model 2w					Model 3w		
				OR^b^ (95% CI)		*P* value		OR (95% CI)		*P* value		OR (95% CI)			*P* value
Intercept^c^				1.40 (−0.04 to 2.84)		.10		−0.64 (−2.04 to −0.77)		.53		−1.19 (−2.51 to 0.12)			.14
**Demographics**															
	Age (years)		1.02 (1.01 to 1.04)		*<.001*		1.02 (1.01 to 1.03)		*.009*		1.01 (1.00 to 1.02)			.*05*	
	Sex (male)		0.97 (0.78 to 1.20)		.76		1.01 (0.82 to 1.23)		.95		0.97 (0.80 to 1.17)			.80	
	**Income (US $)**														
		50,000-75,000	1.19 (0.79 to 1.80)		.60		1.24 (0.84 to 1.83)		.45		0.97 (0.67 to 1.40)			.92	
		75,000-100,000	1.14 (0.75 to 1.74)		.60		1.19 (0.80 to 1.77)		.53		1.01 (0.70 to 1.46)			.96	
		100,000-150,000	1.19 (0.79 to 1.81)		.60		1.38 (0.93 to 2.05)		.23		1.16 (0.80 to 1.68)			.62	
		≥150,000	0.87 (0.56 to 1.35)		.60		1.09 (0.71 to 1.65)		.78		0.92 (0.62 to 1.36)			.79	
	Supervisor (yes)		0.66 (0.54 to 0.81)		*<.001*		0.77 (0.64 to 0.94)		*.03*		0.79 (0.66 to 0.95)			*.04*	
	English (as first language)		1.39 (1.05 to 1.85)		*.05*		1.24 (0.95 to 1.62)		.23		1.07 (0.83 to 1.37)			.78	
	**Education level**														
		College degree	0.86 (0.58 to 1.27)		.60		0.88 (0.61 to 1.27)		.63		0.82 (0.58 to 1.16)			.42	
		Graduate degree	0.88 (0.58 to 1.32)		.60		0.88 (0.60 to 1.30)		.63		0.80 (0.56 to 1.15)			.41	
	**Had a wearable**														
		Unknown	1.60 (1.01 to 2.55)		*.09*		1.33 (0.86 to 2.07)		.35		1.26 (0.83 to 1.91)			.42	
		Yes	1.50 (1.23 to 1.83)		*<.001*		1.33 (1.10 to 1.61)		*.011*		1.20 (1.01 to 1.44)			*.097*	
**Personality**															
	Extraversion		0.67 (0.57 to 0.78)		*<.001*		0.74 (0.64 to 0.86)		*.001*		0.79 (0.69 to 0.91)			*.005*	
	Agreeableness		0.68 (0.56 to 0.83)		*.001*		0.72 (0.60 to 0.87)		*.003*		0.79 (0.66 to 0.94)			*.03*	
	Conscientiousness		1.34 (1.14 to 1.58)		*.001*		1.31 (1.13 to 1.53)		*.003*		1.35 (1.17 to 1.56)			*<.001*	
	Neuroticism		0.85 (0.73 to 0.98)		*.05*		0.85 (0.74 to 0.97)		*.045*		0.85 (0.75 to 0.97)			*.04*	
	Openness		1.03 (0.87 to 1.22)		.75		1.02 (0.87 to 1.20)		.83		0.96 (0.83 to 1.12)			.78	
**Behavior**															
	Survey compliance in week 2 (%)		—^d^		—		1.02 (1.02 to 1.03)		*<.001*		1.01 (1.01 to 1.01)			*<.001*	
	Wearable compliance in week 2 (%)		—		—		—		—		1.02 (1.02 to 1.03)			*<.001*	

^a^*P* values of <.10 are denoted in italics.

^b^OR: odds ratio.

^c^Intercept is not an odds ratio but an estimate.

^d^Variable was not included in the model.

**Table 9:** Model performance.

Test and metrics			Survey compliance							Wearable compliance				
			Model 1s		Model 2s		Model 3s		Model 1w			Model 2w		Model 3w
**Likelihood ratio test**														
	df^a^	19		20		21		19			20		21	
	LogLik	247		405		435		214			283		370	
	Chi-square (*df*)	N/A^b^		316 (1)		59 (1)		N/A			140 (1)		174 (1)	
	*P* value	N/A		<.001		<.001		N/A			<.001		<.001	
**Nonblinded**														
	AIC^c^	−455		−767		−824		−389			−527		−698	
	*R* ^2^	0.14		0.44		0.48		0.19			0.37		0.53	
**5-fold cross-validation**														
	*R*^2^ training set	0.15		0.45		0.49		0.20			0.38		0.54	
	*R*^2^ testing set	0.11		0.44		0.48		0.18			0.33		0.50	
	MAE^d^ training set	0.20		0.14		0.13		0.26			0.22		0.19	
	MAE testing set	0.21		0.14		0.14		0.27			0.23		0.19	
**Blinded**														
	*R* ^2^	0.16		0.58		0.62		0.25			0.50		0.66	
	MAE	0.21		0.12		0.11		0.25			0.20		0.16	

^a^Degrees of Freedom.

^b^N/A: not applicable.

^c^AIC: Akaike information criterion.

^d^MAE: mean absolute error.

### Associations With Compliance

The results in model 1s/1w generally replicated previous findings of associations with compliance: conscientiousness (model 1s: odds ratio [OR] 1.25, 95% CI 1.07-1.46, *P*=.02; model 1w: OR 1.34, 95% CI 1.14-1.58, *P*=.001) and age (model 1s: OR 1.02, 95% CI 1.00-1.03, *P*=.03; model 1w: OR 1.02, 95% CI 1.00-1.02, *P*<.001) were positively associated with compliance [13,31,34], sex did not have an effect on compliance [31], and extraversion had a negative effect (model 1s: OR 0.74, 95% CI 0.64-0.85, *P*<.001; model 1w: OR 0.67, 95% CI 0.57-0.78, *P*<.001) in compliance [31]. However, agreeableness (model 1w: OR 0.68, 95% CI 0.56-0.83, *P*=.001) and neuroticism (model 1w: OR 0.85, 95% CI 0.73-0.98, *P*=.05) were negatively associated with compliance, and income, education, and openness were not found to be statistically significant, which contradicts previous studies [13,31,33,34], possibly because of the following methodological differences: we treated compliance as a ratio and not a binary variable, our models included more controls than previous studies, and our sample was one of information workers, not of students, that also under sampled lower-income workers.

English as a native language was associated with higher compliance in our sample (model 1s: OR 1.38, 95% CI 1.05-1.80, *P*=.06; model 1w: OR 1.39, 95% CI 1.05-1.85, *P*=.05). Command of the English language could have facilitated participation in our study, given how all manuals, surveys, and communications with participants were written in English. Having a supervisory role, which we speculated could be an indicator of busyness, was negatively associated with compliance as expected. Participating in the study is one more competing need in a busy schedule that could preclude participants from dedicating as much time as they would have done otherwise. Finally, having had a wearable was positively associated with compliance (model 1s: OR 1.25, 95% CI 1.04-1.51, *P*=.06; model 1w: OR 1.50, 95% CI 1.23-1.83, *P*<.001). People who have had wearables before were familiar with the technology and demonstrated interest in them, which could serve as motivation to use it more, thus staying more compliant.

The significance of age, supervising role, and having had a wearable changed in the models as early compliance variables were added, possibly because of early compliance being highly correlated with long-term compliance (Table 2), and these other variables not helping above and beyond early compliance as controls.

### RQ1: Participant Characteristics and Compliance Before the Study Starts

When it comes to goodness of fit, the results from model 1s and model 1w show that with a demographics and personality survey, we can explain 19% of the wearable compliance variance (model 1w) and 14% of the daily survey compliance variance (model 1s). Cross-validated and blinded set *R*^2^ shows that the models are not substantially overfitting with *R*^2^ values close (15% versus 11% and 20% versus 18%) or slightly higher (14% versus 16% and 19% versus 25%) than the values for the data used in fitting the model (training versus testing and nonblinded versus blinded). Cross-validated results show a testing set MAE of 0.21 (model 1s) and 0.27 (model 1w), indicating that when using 80% of the data for training and predicting on new data, the predictions are on average within 0.21 and 0.26 of the actual compliance. The correlation between the predicted and actual values slightly decreases when comparing training and testing sets, which is expected. However, the MAE also indicates that the model is not substantially overfitting (even when training on 80% of the data) as the error increase from training to testing is ≤0.01.

In the blinded set, the error of model 1s was similar to that of the cross-validated version when comparing MAE (0.21). In the case of model 1w, the MAE was lower in the blinded set (0.20 versus 0.23).

### RQ2: Early Assessment of Compliance and Long-term Compliance

We constructed models 2s/2w and 3s/3w to address whether very early compliance would be indicative of future compliance, as compliance has been shown to be trait-like [12,31,74]. Early daily survey compliance in week 2 was a good predictor for both survey and wearable compliance (model 2s: OR 1.03, 95% CI 1.03-1.04, *P*<.001; model 2w: OR 1.02, 95% CI 1.02-1.03, *P*<.001), and thus a good proxy for trait-like compliance.

Models 2s/2w and 3s/3w show a significant improvement when compared with the corresponding model 1. Adding early wearable compliance in model 3s/3w improved the fit across all tests and provided an improvement in our blinded data set by reducing error. As more tasks are added, more trait-like compliance can be captured early on. A lower Akaike information criterion indicated that both models are of better relative quality than the corresponding model 1, and models 3s/3w are better than models 2s/2w. Likelihood ratio tests further confirmed this. Although a training increase in *R*^2^ is expected in regression models as more variables are added, the improvement on the cross-validated and blinded set *R*^2^ with respect to the respective model 1 values shows a far better fit to previously unseen data.

When comparing model 3s with model 2s in predicting survey compliance, the benefit of including wearable compliance in week 2 (model 3s: OR 1.01, 95% CI 1.01-1.01, *P*<.001) is minor, with only a small increase in fit and a decrease in error. In the case of predicting wearable compliance, model 3w shows an improvement over model 2w by including wearable compliance in week 2 (model 3w: OR 1.02, 95% CI 1.02-1.03, *P*<.001). Cross-validated MAE reduced by 0.04, and *R*^2^ increased by 0.17, whereas the blinded set *R*^2^ increased by 0.16, and MAE decreased by 0.04.

Overall, survey compliance can be predicted with less error than wearable compliance. The cross-validated and blinded set MAE of both predictions is within a reasonable value to be useful, and the relatively high *R*^2^ indicates that the predictions correlate highly with actual compliance.

Finally, as we predicted aggregated compliance, we needed to make sure that compliance is relatively stable to make sure that the predictions of these models could be generalized to studies of varying lengths. We know from Table 2 that week 2 compliance and long-term compliance are correlated. Therefore, we calculated intraclass correlation coefficients (ICC) and found that there was good agreement [75] between weekly compliances throughout the study in the entire data set (blinded and nonblinded) for both variables: wearable compliance, ICC was 0.695 (95% CI 0.673-0.718), and survey compliance, ICC was 0.656 (95% CI 0.630-0.682). Furthermore, to test the agreement of aggregate measures when surveys were no longer required, we aggregated wearable compliance until day 56 and correlated it with study-long compliance showing good agreement as well *r*=.82 (95% CI 0.80-0.85). The correlations in Table 2—ICCs—along with previous findings [31], suggest that any long-term aggregate of compliance would significantly correlate with the aggregation of compliance used in the analyses (56 days for surveys and year-long for wearable).

### RQ3: Self-assessment of Compliance

Among the participants who answered our questions related to compliance and study participation, 80.9% (466/576) thought that it was easy to participate in the study, 75.9% (438/577) thought that they were extremely well- or somewhat well-compensated, and 95.1% (548/576) said that we could contact them if we were to run another study like this one. Notably, 65.1% (375/576) of the participants agreed that the portal was useful, 17.4% (100/576) neither agreed nor disagreed that the portal was useful, and only 17.5% (101/576) found the portal not useful or did not recall using it. 84.5% (484/573) of the participants felt somewhat or extremely satisfied with the way issues were resolved throughout the study. Overall, participants found that the biggest obstacles toward compliance were technical issues (280/555, 50.5%), followed by personal reasons (203/555, 36.6%), work reasons (107/555, 19.2%), survey issues (75/555, 13.5%), difficulty with setup (48/555, 8.6%), privacy (20/555, 3.6%), and other reasons (63/555, 11.4%).

In addition, portal use and perceptions were correlated with compliance. Portal usefulness was positively correlated with wearable compliance (*r*=0.139; *P*=.002) and survey (*r*=0.217; *P*<.001) compliance. Perceived timely resolution was correlated with wearable compliance (*r*=0.250; *P*<.001) and survey compliance (*r*=0.128; *P*=.002). Ease of study participation was associated with wearable compliance (*r*=0.355; *P*<.001) and survey compliance (*r*=0.252; *P*<.001). Finally, compensation was positively correlated with wearable compliance (*r*=0.281; *P*<.001) and survey compliance (*r*=0.190; *P*<.001). Taken together, these correlations demonstrate that, in general, perceived usefulness, timely issue resolution, ease of study participation, and adequate compensation are associated with higher compliance. Thus, effective feedback and timely problem resolution are useful goals for researchers looking to maximize compliance.

## Discussion

### Recommendations for Future Study Design

Although researchers cannot change the characteristics of participants found to be associated with compliance (ie, demographics and personality), there are still different strategies based on the models presented throughout this work and the exploration of the RQs proposed.

#### Oversampling

One initial strategy could be oversampling groups likely to drop out or be less compliant (eg, those who had higher extraversion or lower conscientiousness). We do not recommend simply excluding participants based on these variables, as this would introduce bias. We showed that a short survey of demographics and personality traits could predict compliance early on, with an MAE of 0.26 for wearable compliance (model 1w) and 0.20 for daily survey compliance (model 1s). Furthermore, our findings related to RQ2 suggest that there is value in an initial 2-week pilot in which participants fill out a subset of actual tasks or rough equivalents. We do not think the items themselves matter but only that the surveys are short (<2 minutes to complete). We would expect this to generalize as follows: completion of *some* of the full set of study tasks during the pilot will allow researchers to observe a *trait-like* characteristic of compliance in the participants, which will be indicative of how much they comply with study tasks in general. Alternatively, the pilot could include the full set of tasks (eg, survey completion and wearable use). This proved to have the best fit in our models for both kinds of compliance studied. However, in the case of a study involving wearables, providing the devices to the pilot participants could entail higher costs with minimal benefit (Table 9; models 3s/3w versus model 2s/2w). Nevertheless, a 2-week pilot would be cost-effective for studies that pay more as more data get collected or studies that require a certain level of data regardless of the population being sampled. Using Tesserae payment as an example, participants were paid US $50 to complete enrollment at week 1, US $150 at the end of week 12, US $200 at the end of 24 weeks, and US $350 at the conclusion of the study. A total of 107 participants dropped out of the study. In many study designs, data from participants who do not complete the study might be excluded because of insufficient data. If all 107 participants were compliant through week 12 and subsequently became noncompliant or dropped out, participants would have been paid US $200 for data that may not be useful in achieving the study goals. If a survey-only pilot lasting 2 weeks was conducted that screened 107 noncompliant participants, they would only have cost the study US $50. With the US $150 savings per participant, plus the savings of US $550 not paid to participants who dropped out, Tesserae researchers could have recruited an additional 99 fully compliant participants without deviating from the original budget. Researchers who have a specific budget for participant payments can thus maximize data collection by estimating compliance through a short pilot study.

#### Targeted Participant Engagement

If a study is not long enough or the budget does not allow for oversampling, using the models early could suggest to researchers which participants are likely to require extra support to engage with them properly or perhaps provide flexibility or an adaptive schedule in the completion of tasks, such as EMAs that interrupt ongoing activities unlike passive sensing [37]. Approximately 14% of the participants who answered what the biggest difficulties in maintaining compliance were identified *surveys too often, too long, and too many* as one of their difficulties. However, researchers need to carefully consider the interventions in the study to prevent them from being counterproductive. Throughout our study, the interventions were kept to a minimum. Participants were reminded of syncing their wearable only once a week (Mondays) in the event of missing data (most likely because of delayed data syncing from the wearable) and only if initial data from the wearable had not been received. Similar interventions were sent in the case of missing smartphone agent data, lack of beacon sightings in a significant period, or consistent periods of not responding to daily surveys. Despite limited interventions, the compliance for the study was quite high, with median compliance rates of 85.7% (649/757) for the daily surveys, 84.7% (641/757) for the wearables, and 93.7% (709/757) for the smartphone agent among all participants. Obtaining adequate compliance with minimal interventions can reduce experimenter effort and save experimenter resources while reducing participant interaction. More interventions do not necessarily produce better compliance [19,31], and too many notifications can lower their importance to participants [76].

#### Providing a Study Portal

Finally, we recommend providing support in the form of troubleshooting and a compliance tracking portal that can help participants stay compliant [77]. The portal in our study comprised an issue tracker and dashboards for the researchers and participants, with study researchers being able to track compliance as a study aggregate as well as per participant. Participants were able to track their compliance throughout the study and easily contact the researchers in cases where they saw a discrepancy between the compliance levels shown on the portal and their own expectations. Participants confirmed the usefulness of the portal, with 65.1% (375/576) believing that it was useful and only 17.5% (101/576) thinking that it was not useful or not remembering having used it.

### Limitations and Future Work

It is important to note the several limitations of this work. As the participants were largely drawn from a population of information workers from 4 organizations, these participants may not represent all information workers or the general working population. For example, lower-income individuals were underrepresented in the sample. Although ethnicity was previously not found to be associated with compliance [31], we did not collect information about race; thus, we do not have a way of knowing if the sample was diverse and representative of the broader US population.

In addition, the study design could not explore how maintaining compliance on one stream affected compliance for other streams. Thus, it is possible that a 2-week pilot with only surveys may not have the same effect on compliance as a 2-week pilot with all streams.

Finally, there is the aspect of rewards and compensation that we could not examine or control for in our analysis because of study requirements and the fact that all participants that received lottery payments belonged to a single cohort. Although Musthag et al [78] found no differences among the 3 payment schemes in a study comparing the effect of incentives on compliance, the authors believed that if the incentives had lower values, they would have observed differences in the compliance rate. Harari et al [33] found a markedly different compliance across the 3 incentive schemes that relied on course credit and feedback, compensation and feedback, and a prize reward—keeping the wearable—at the end of the study. Given that at least 9.9% (57/577) of our participants found compensation to be inadequate, with a slight majority (35/57, 61%) of that 9.9% having received lottery payments, it is possible that a future study would find stipend compensation to be more effective than lottery-based payments.

In addition to addressing the above limitations, future work could examine the rate of dropouts through a survival analysis using time-varying covariates, as well as whether periods or onset of noncompliance are marked by spikes in stress or changes in sleep patterns. In this work, as we focused on the early prediction of long-term compliance instead of ongoing prediction, we did not include time-varying covariates. Furthermore, developing purely predictive models based on the findings of this work and such time-series analyses could lead to the development of effective study design and management tools that support decisions before and during the study to maximize compliance in studies.

### Conclusions

Our work is an extensive analysis of sensor compliance for a longitudinal study of a population of information workers from multiple organizations and across the United States. We presented predictions of compliance in the Tesserae study along with a detailed description of the methodology of the study. We considered 31 variables and presented 6 beta regression models, with 14 selected variables that evaluated the association between compliance and participants’ demographics, personality, and trait-like compliance early in the study. We presented participants’ challenges in maintaining compliance, their satisfaction with troubleshooting issues, and their assessment of the portal that provided feedback on compliance and help with troubleshooting. From this work, we draw recommendations for future longitudinal studies that aim to improve efficiency by maximizing the amount of data collected. Ultimately, our work provides insights to improve the experimental setup of a study to maximize the quantity of data collection.

## Appendix

Multimedia Appendix 1 Other variables collected for exploratory analysis.

Multimedia Appendix 2 Exploratory analysis.

## Abbreviations

EMA: ecological momentary assessment
IARPA: Intelligence Advanced Research Projects Activity
ICC: intraclass correlation coefficient
MAE: mean absolute error
OR: odds ratio
RQ: research question
