# Supplementary material for: Predicting Participant Compliance With Fitness Tracker Wearing and Ecological Momentary Assessment Protocols in Information Workers: Observational Study
Source: JMIR Mhealth Uhealth. 2021 Nov 12;9(11):e22218. doi: 10.2196/22218 (PMC8663716; doi:10.2196/22218)
Supplement: Multimedia Appendix 1 [file mhealth_v9i11e22218_app1.docx]

## Other variables collected for exploratory analysis

Table S1. Inventories used to assess Affect, Anxiety, Cognitive Ability, Health, and Job Performance.

| Category | Inventories used for assessment | Questions |
| --- | --- | --- |
|  |  |  |
| Affect | Positive and Negative Affect Schedule Expanded [47] (PANAS-X)^a^ | 60 |
| Anxiety | State-Trait inventory [46] | 20 |
| Cognitive Ability | Shipley 2 inventory [53] | 65 |
| Health | Tobacco use assessed by a modified Global Adult Tobacco Survey [48] (GATS) | 3 |
|  | Alcohol use assessed by The Alcohol Use Disorders Identification Test [49] (AUDIT) | 10 |
|  | Physical activity assessed by the International Physical Activity Questionnaire [50,51] (IPAQ) | 27 |
|  | Sleep Quality assessed by The Pittsburgh Sleep Quality Index [52] (PSQI) | 19 |
| Job Performance | In Role Behavior [54] (IRB) | 7 |
|  | Individual Task Proficiency [55] (ITP) | 3 |
|  | Interpersonal and Organizational Deviance [56] | 19 |
|  | Organizational Citizenship Behavior [57] (OCB) | 20 |
| Personality | Big Five Inventory [45] (BFI)^b^ | 60 |

^a^ Participants were asked about how they felt *on average*.

^b^ The scale used is from 1 to 5 and they represent the mean of the responses to the questions assessing each personality trait.

Figure S1: Distribution of other variables^a^ in the Non-Blinded dataset.


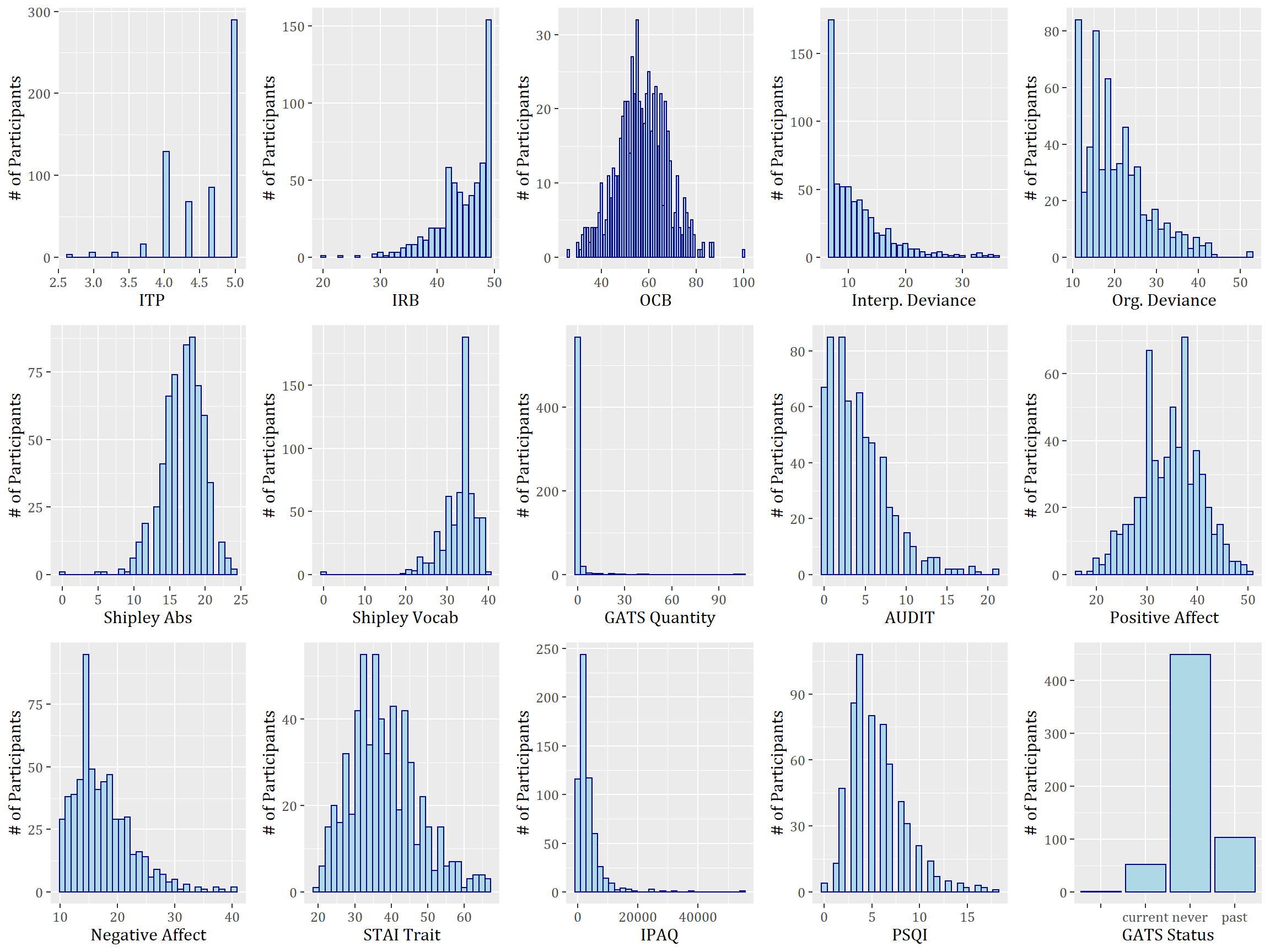


^a^AUDIT: Abbreviations: Alcohol Use Disorders Identification Test. GATS: Global Adult Tobacco Survey. IPAQ: International Physical Activity Questionnaire. IRB: In-Role Behavior. ITP: Individual Task Proficiency. OCB: Organizational Citizenship Behavior. PSQI: Pittsburgh Sleep Quality Index. STAI: State-Trait Anxiety Inventory.
