# Supplementary material for: Predicting Participant Compliance With Fitness Tracker Wearing and Ecological Momentary Assessment Protocols in Information Workers: Observational Study
Source: JMIR Mhealth Uhealth. 2021 Nov 12;9(11):e22218. doi: 10.2196/22218 (PMC8663716; doi:10.2196/22218)
Supplement: Multimedia Appendix 2 [file mhealth_v9i11e22218_app2.docx]

## Exploratory Analysis

### Overview

In Table 11, we show models predicting survey and wearable compliance with all possible predictors except early compliance fitted with the non-blinded dataset (N=596 due to one additional participant having missing data). No correction was applied to the *P* values in this model to avoid incurring Type 2 errors as most of the new variables in these models have not been studied in relation to compliance before. However, note that more of these findings may be due to chance.

In our exploratory analysis, we found several predictors that were associated with compliance that were not reported in the main text: Education (College Degree vs Highschool Degree, Graduate Degree vs Highschool Degree), IPAQ, PSQI, Positive Affect, Fluid Cognitive Ability (Shipley Abstract), Interpersonal Deviance, Number of Issues in Week 2, and Number of Logins in Week 2. Adding these variables to the models did not yield a model that reduced the prediction error in the non-blinded dataset over Models 3s/w. However, researchers could consider these variables for future study. We discuss the possible relationships of the variables that have seldom been studied in relation with compliance.

### Cognitive Ability

Using the Shipley-2 test, we measured fluid and crystallized cognitive ability. While crystallized cognitive ability reflects learned or acquired abilities, fluid cognitive ability reflects an ability to reason and adapt to new challenges. Participants that score higher in Shipley Abstraction (fluid cognitive ability) are better at solving problems that cannot typically be solved by relying on previously acquired knowledge. For this study, it is possible that higher scores translated into higher wearable and survey compliance because fluid intelligence is useful for solving technical issues arising from the wearable and making the new experience of participating in a study more accessible.

### Interpersonal Deviance

To the best of our knowledge, interpersonal deviance has not been associated with compliance in previous works. However, interpersonal deviance has been positively related to the responsiveness/sensitivity to rewards as found by Diefendorff and Mehta [69]. If the compensation/reward in a study is a good motivator, people with higher interpersonal deviance should be more motivated by it, possibly eliciting a higher study participation. Therefore, it is possible that participants that score higher in interpersonal deviance could have greater compliance than those who score lower.

### Number of Issues and Logins

It is difficult to discern whether the significance of the positive relationship with compliance exists due to the imbalance in the dataset or because there is a real effect through which those that engaged with researchers early were more likely to achieve higher survey and wearable compliance later on, or if the variable captured a trait-like aspect of communication (willingness or readiness to ask for help) or attention to detail to notice discrepancies between perceived and reported compliance. However, it is important to note the fact that the number of issues is associated with compliance when controlling for the number of logins, i.e. not only checking on the portal but reporting differences, which could point to the number of issues measuring a trait or willingness to engage with the study early on in addition to the logins.

### Physical Activity

The IPAQ inventory asks participants about weekly duration in a series of physical activities. Inactivity has been associated with decreased use of activity trackers in general [70]. Rowlands et al. [12] found that physical activity was positively associated with wearing time. Therefore, we would have expected those who were more physically active according to the IPAQ inventory to have been more motivated to wear the fitness tracker in the study. However, we found the opposite. Scoring higher in the IPAQ inventory was associated negatively with wearable compliance (no association found with survey compliance).

### Affect

A measurement of positive affect indicates the extent to which a person feels enthusiastic, energetic, confident, active, alert. It is also related to having healthier coping mechanisms, being more open-minded, sociable, and being better at creative problem solving [71,72]. Higher levels of negative affect, which is independent of positive affect, relate to higher levels of distress, and the experience of negative emotions such as anger, contempt, disgust, guilt, fear, and nervousness [73]. To the best of our knowledge, neither positive nor negative affect have been reported to be associated with compliance in either EMA or wearable studies before. However, affect has been linked to reward/punishment mechanisms that could underpin reward-based compliance. Negative affect decreases expectations of reward and increases expectations of punishment [74], and can translate into pessimism and distrust rather than optimism and expectations of succeeding [75]. Conversely, positive affect has been found to improve motivation. Erez and Isen [76] reported that positive mood inducted participants performed better, exhibited more persistence, tried harder, and reported higher levels of motivation when solving anagrams compared to those who underwent neutral mood induction. Considering that positive and negative affect have been related in other studies to motivation and the expectation of rewards and punishment, it is possible they could be associated with compliance. In our models, there is a negative association between positive affect and compliance. This finding warrants further study as we would have expected participants with higher positive affect to be more motivated to participate in the study.

### Tobacco Consumption

Tobacco and compliance findings in the literature are conflicted, with Lee et al. finding non-smokers to be more compliant[13] and Schüz et. al. [22] finding no effect. Our results align with those of Lee et al. [13] as we found a negative association between compliance and tobacco consumption (currently being a smoker) after controlling for all other variables in our models.

**Sleep Quality (PSQI)**

To measure sleep quality, the study used the PSQI inventory where a lower score means higher sleep quality. The inventory can serve to separate “good sleepers” from “poor sleepers”. A participant with a score greater than 5 is considered a poor sleeper[52]. There is a great body of research regarding the effects of sleep quality in multiple areas of daily life such as job performance, cognitive functioning, and mental health [77,78]. Higher sleep quality meant higher survey and wearable compliance in our exploratory analysis. We think that in the same way that sleep disturbances can affect the previously mentioned areas of daily life, they can result in lower compliance. Lower compliance could be related to sleep disturbances causing lack of mental focus and a more difficult learning experience. It would be feasible to think that mental errors such as forgetting to wear the device could happen more often due to sleep disturbances.

Table 11: Exploratory models of compliance. *P* values < .1 are highlighted in italics.

| Category and Variables | | Survey Compliance^a^ | | | Wearable Compliance^b^ | | |
| --- | --- | --- | --- | --- | --- | --- | --- |
|  | | OR | 95% CI | *P* | OR | 95% CI | *P* |
|  | | | | | | | |
| Intercept^c^ | | 0.13 | -1.97, 2.22 | .91 | 0.33 | -1.89, 2.54 | .77 |
| **Demographics** | | | | | | | |
|  | Age | 1.02 | 1.01, 1.03 | *.001* | 1.03 | 1.02, 1.05 | *<.001* |
|  | Sex (Male) | 1.07 | 0.87, 1.31 | .53 | 0.89 | 0.72, 1.10 | .29 |
|  | Income 50k to 75k | 0.96 | 0.65, 1.41 | .82 | 1.05 | 0.69, 1.58 | .83 |
|  | Income 75k to 100k | 0.86 | 0.58, 1.28 | .46 | 0.99 | 0.65, 1.51 | .97 |
|  | Income 100k to 150k | 0.78 | 0.52, 1.15 | .21 | 0.94 | 0.62, 1.42 | .76 |
|  | Income 150k or higher | 0.61 | 0.40, 0.93 | *.02* | 0.70 | 0.45, 1.09 | .11 |
|  | Supervisor (Yes) | 0.68 | 0.56, 0.84 | *<.001* | 0.71 | 0.57, 0.88 | *.002* |
|  | English (as First Language) | 1.31 | 0.97, 1.77 | *.08* | 1.40 | 1.02, 1.93 | *.04* |
|  | Education Level (College Degree) | 0.86 | 0.59, 1.26 | .45 | 0.71 | 0.48, 1.06 | *.094* |
|  | Education Level (Graduate Degree) | 1.02 | 0.68, 1.52 | .93 | 0.68 | 0.44, 1.03 | *.07* |
|  | Had a Wearable (Unknown) | 1.50 | 0.97, 2.32 | *.07* | 1.70 | 1.08, 2.69 | *.02* |
|  | Had a Wearable (Yes) | 1.16 | 0.96, 1.41 | .12 | 1.39 | 1.14, 1.70 | *.001* |
| **Personality** | | | | | | | |
|  | Extraversion | 0.80 | 0.68, 0.94 | *.008* | 0.77 | 0.64, 0.92 | *.003* |
|  | Agreeableness | 0.94 | 0.77, 1.14 | .52 | 0.73 | 0.59, 0.89 | *.002* |
|  | Conscientiousness | 1.33 | 1.12, 1.58 | *.001* | 1.52 | 1.27, 1.82 | *<.001* |
|  | Neuroticism | 0.98 | 0.80, 1.20 | .86 | 0.79 | 0.64, 0.98 | *.03* |
|  | Openness | 1.05 | 0.89, 1.23 | .59 | 1.04 | 0.87, 1.23 | .69 |
| **Anxiety, Health and Affect** | | | | | | | |
|  | STAI Trait | 1.00 | 0.98, 1.02 | .79 | 1.02 | 1.00, 1.04 | .13 |
|  | GATS Status – Past | 0.94 | 0.73, 1.21 | .65 | 0.94 | 0.72, 1.22 | .63 |
|  | GATS Status - Current | 0.70 | 0.48, 1.01 | *.06* | 0.89 | 0.60, 1.33 | .58 |
|  | GATS Quantity | 1.00 | 0.99, 1.02 | .71 | 1.00 | 0.98, 1.01 | .63 |
|  | AUDIT | 1.01 | 0.98, 1.03 | .63 | 1.00 | 0.97, 1.03 | .87 |
|  | PSQI | 0.96 | 0.93, 0.99 | *.02* | 0.95 | 0.91, 0.98 | *.004* |
|  | IPAQ | 1.00 | 0.98, 1.02 | .79 | 0.97 | 0.95, 0.98 | *.006* |
|  | Positive Affect | 0.98 | 0.96, 1.00 | *.06* | 0.99 | 0.97, 1.02 | .53 |
|  | Negative Affect | 0.98 | 0.96, 1.01 | .14 | 0.99 | 0.97, 1.02 | .60 |
| **Cognitive Ability and Work Performance** | | | | | | | |
|  | Shipley Abstract | 1.04 | 1.01, 1.08 | *.02* | 1.08 | 1.04, 1.13 | *<.001* |
|  | Shipley Vocabulary | 1.00 | 0.98, 1.03 | .85 | 1.00 | 0.97, 1.03 | .81 |
|  | Individual Task Performance | 0.98 | 0.78, 1.23 | .87 | 0.82 | 0.65, 1.05 | .11 |
|  | In-Role Behavior | 1.01 | 0.98, 1.03 | .60 | 1.00 | 0.97, 1.02 | .90 |
|  | Organizational Citizenship Behavior | 1.00 | 0.99, 1.01 | .45 | 0.99 | 0.98, 1.00 | .28 |
|  | Organizational Deviance | 1.00 | 0.98, 1.01 | .62 | 1.00 | 0.98, 1.01 | .59 |
|  | Interpersonal Deviance | 1.03 | 1.01, 1.05 | *.005* | 1.02 | 1.00, 1.04 | *.093* |
| **Behavior** | | | | | | | |
|  | Number of Issues in Week 2 | 1.23 | 1.04, 1.45 | *.09* | 1.22 | 1.02, 1.45 | *.03* |
|  | Number of Logins in Week 2 | 1.09 | 0.99, 1.20 | *.01* | 1.04 | 0.94, 1.15 | .49 |

^a^Survey compliance R^2^ = .21

^b^Wearable compliance R^2^ = .28

^c^Intercept is not an odds ratio but an estimate.
